# Pleural effusion revealing a ruptured mediastinal mesothelial cyst

**DOI:** 10.1002/rcr2.659

**Published:** 2020-09-08

**Authors:** Ahmed Ben Saad, Saousen Cheikh Mhamed, Asma Migaou, Asma Achour, Naceur Rouatbi, Samah Joobeur

**Affiliations:** ^1^ Pulmonology Department Fattouma Bourguiba Teaching Hospital Monastir Tunisia; ^2^ Radiology Department Fattouma Bourguiba Teaching Hospital Monastir Tunisia

**Keywords:** Mediastinum, mesothelial cyst, pleural effusion, video‐assisted thoracoscopy

## Abstract

Mesothelial cysts are uncommon benign cysts of the mediastinum. Rarely, they are discovered after complications or unusual presentations. This report describes a rare case of pleural effusion revealing a ruptured mediastinal mesothelial cyst in a 28‐year‐old man. The diagnosis of this complicated mesothelial cyst relied on intraoperative and anatomopathological findings. He underwent videothoracoscopy with resection of the cyst. Outcomes were favourable.

## Graphical abstract

We report a rare case of a mediastinal mesothelial cyst discovered after rupture in the pleural cavity in a 28‐year‐old man. The diagnosis of this complicated mesothelial cyst relied on intraoperative and anatomopathological findings. To the best of our knowledge, we report the first case of a pleural effusion revealing a ruptured mediastinal coelomic cyst into the pleural cavity.

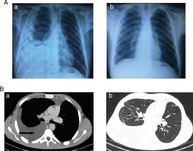

## Introduction

Mesothelial or coelomic cysts are uncommon unilocular benign cysts of the mediastinum [1, 2]. Rarely, they are discovered after complications or unusual presentations [2, 3, 4]. Here, we describe a rare case of a mediastinal mesothelial cyst discovered after rupture in the pleural cavity.

## Case Report

**Figure 1:**
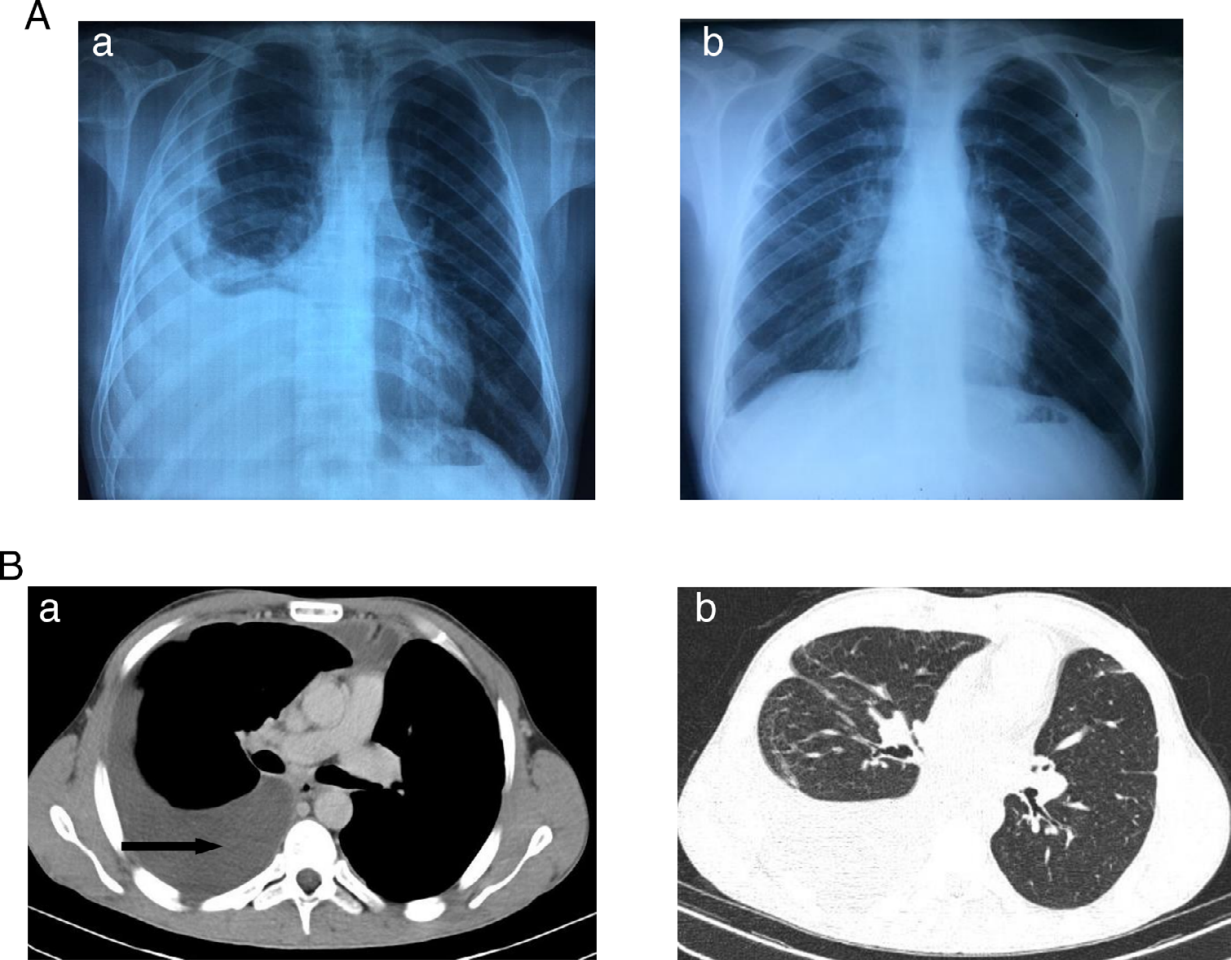
Imaging findings. (A) Frontal chest radiograph. a, Before treatment: an opacity of the lower part of the right hemithorax corresponding to a pleural effusion. b, After video‐assisted thoracoscopic surgery (VATS): no recurrence of pleural effusion. (B) Chest computed tomography (CT) scan: (axial image—a: mediastinal and b: parenchymal windows): a right‐sided pleural effusion (black arrow) without evidence of pleural or parenchymal lesion.

**Figure 2:**
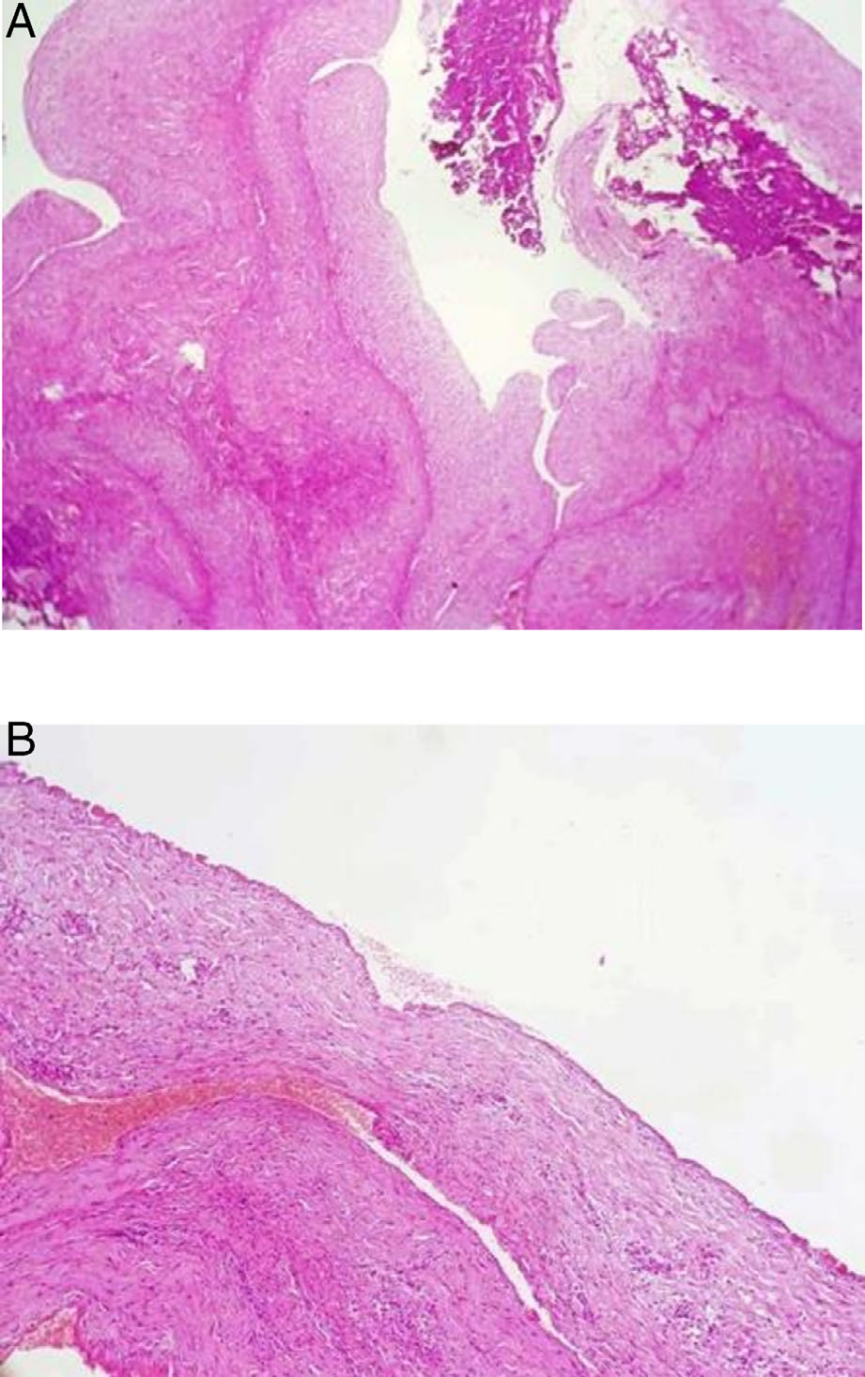
Pathological examination of the cyst (A: haematoxylin and eosin (HE), 40× and B: HE, 100×): Thin‐walled cyst lined by single layer of non‐pleomorphic cells.

A 28‐year‐old man with an uneventful past medical history admitted to our department for chest pain and dyspnoea that started two days ago. He had six pack‐year smoking history. He was not complaining of any other sign. The physical examination was in favour of the diagnosis of a right‐sided pleural effusion. Chest X‐ray showed an opacity of the lower part of the right hemithorax (Fig. 1A‐a). The pleural puncture was performed showing a clear pleural fluid with high lymphocytosis at 75% and protein level at 56 g/L. The search for tuberculosis bacillus in the pleural fluid and in sputum was negative. No tumour cells were found in the pleural fluid. Fibre optic bronchoscopy revealed an extrinsic compression on the right lower lobe bronchus. Abrams pleural biopsy demonstrated a non‐specific inflammation. Chest computed tomography (CT) scan showed a right‐sided pleural effusion without evidence of pleural or parenchymal lesion (Fig. 1B). The patient underwent a videothoracoscopy. Intraoperative findings revealed an anterior mediastinal thin‐walled cystic structure ruptured into the pleural cavity. The resection of the latter was performed. Anatomopathological examination demonstrated a mesothelial cyst (Fig. 2). One year after the intervention, the patient is asymptomatic without any anomaly at the chest X‐ray (Fig. 1A‐b) or control CT scan.

## Discussion

Coelomic cysts are derived mainly from the pericardium. They are due to embryonic tissue development abnormalities [4]. The cyst is surrounded by a single layer of mesothelial cells wall and contain clear fluid [3]. Mediastinal cysts are often asymptomatic [1]. Symptoms such as cough, dyspnoea, or chest pain can be seen by compression of adjacent cardiovascular or respiratory structures [1].

Atypical and rare presentations are described in the English literature including rupture, migration, or uncommon sites. In fact, coelomic cysts arising from the extramediastinum are extremely rare. Only two cases of mesothelial cyst deriving from the chest wall pleura were reported [3]. Walker et al. described an atypical presentation of a migrating coelomic cyst into the major fissure, attached to the pericardium in an asymptomatic 45‐year‐old woman [4]. Only two cases of migrant mesothelial cysts have been reported [4]. The rupture of a mediastinal mesothelial cyst into the pericardium causing tamponade was reported by El Hammoumi et al. [5]. To the best of our knowledge, we report the first case of a pleural effusion revealing a ruptured mediastinal coelomic cyst into the pleural cavity. The rupture of the cyst into the close structure can be caused by either erosion, high intracystic tension, infection, or trauma [2, 5]. The cause of rupture of the cyst in our case may be due to high intracystic tension with erosion of adjacent structures. In fact, no evidence of chest trauma or clinical signs of infection was found. The mechanism of the formation of the large exudative pleural effusion is controversial. Some mesothelial cysts can present a rapid and important growth leading to a voluminous cyst. Furthermore, the irritation of the pleura after the rupture of the cyst may contribute to this exudative pleural reaction. The hypothesis of a pleural infection is unlikely because of the absence of clinical signs of infection and the low proportion of neutrophils in the pleural fluid.

Chest CT scan allows diagnosis by showing a fluid‐filled, thin‐walled cyst without enhancement after contrast injection [2]. In case of a ruptured cyst, the diagnosis is more difficult. No consensus on the coelomic cyst treatment exists. Owing to its benign behaviour, some authors recommend simple surveillance unless the diagnosis is uncertain or there are complications [1]. Mini‐invasive procedures like video‐assisted thoracoscopic surgery (VATS) currently represent the best approach [1]. However, the excision of the cyst does not prevent from a possible recurrence [3].

In conclusion, despite being usually asymptomatic, mediastinal mesothelial cyst can be discovered after complications. The rupture in the pleural cavity is an extreme rare condition and imaging can fail to make the diagnosis. VATS is the best therapeutic approach even for complicated cysts.

### Disclosure Statement

Appropriate written informed consent was obtained for publication of this case report and accompanying images.
